# Assessment of Carotid Atherosclerosis in Type 2 Diabetes Mellitus Patients with Microalbuminuria by High-Frequency Ultrasonography

**DOI:** 10.1155/2013/819584

**Published:** 2013-03-14

**Authors:** Yu-Hong Zhang, Yuan Gao, Xin Mao, Jing Shang, Ben-Li Su

**Affiliations:** ^1^Department of Diagnostic Ultrasound, Second Affiliated Hospital of Dalian Medical University, 467 Zhongshan Road, Dalian 116023, China; ^2^Department of Diagnostic Ultrasound, Xin Hua Hospital of Dalian University, Dalian 116023, China; ^3^Department of Endocrinology, Second Affiliated Hospital of Dalian Medical University, Dalian 116023, China

## Abstract

The aim of this study is to evaluate carotid atherosclerosis in patients of type 2 diabetes mellitus with microalbuminuria (MA) by high-frequency ultrasonography. Two hundred and fifty patients of type 2 diabetes mellitus were divided into two groups according to urinary albumin excretion rate (UAER): normoalbuminuria group (130 cases) and microalbuminuria group (120 cases). The intimal-medial thickness (IMT) and the atherosclerotic plaques of carotid artery were observed in both groups by high-frequency ultrasound. Fasting blood glucose (FBG), hemoglobin A1c, and lipid profiles were measured. The values of IMT of microalbuminuria group were significantly higher than those of normoalbuminuria group (*P* < 0.05). In univariate analysis, IMT was positively and significantly associated with age (*r* = 0.265, *P* < 0.05), waist circumference (*r* = 0.263, *P* < 0.05), body mass index (*r* = 0.285, *P* < 0.05), systolic blood pressure (*r* = 0.276, *P* < 0.05), UAER (*r* = 0.359, *P* < 0.05), HbA1c (*r* = 0.462, *P* < 0.05) and, duration of diabetes (*r* = 0.370, *P* < 0.05). In multivariate linear regression analysis, UAER and HbA1c were independent predictors of IMT (*P* < 0.05 for all). In the two groups, the rate of soft plaques was higher than that of dense plaques and calcified plaques. In conclusion, there is a significant association between microalbuminuria and IMT which is regarded as the early sign of carotid atherosclerosis in type 2 diabetic patients.

## 1. Introduction

Diabetes mellitus is associated with aggressive vascular abnormalities in human subjects, and atherosclerosis is regarded as the leading cause of morbidity and mortality in diabetic patients [1]. Microalbuminuria has a strong prediction of both the development of diabetic nephropathy and subsequent atherosclerotic vascular dysfunction [2, 3]. The previous epidemiologic study has proved the predictive value of microalbuminuria for atherosclerotic vascular disease in the patients of type 2 diabetes [4]. Several biochemical parameters including soluble vascular cell adhesion molecule1, sialic acid, C-reactive protein, and fibrinogen have been proved to be significantly associated with microalbuminuria [5–7]. And those parameters were believed to indicate endothelial dysfunction and chronic inflammation. These findings may support a hypothesis that microalbuminuria reflects generalized vascular damage which may promote atherosclerosis [8, 9]. But the underlying mechanism is still unclear. High-frequency B-mode ultrasonography is a noninvasive method of detecting carotid artery wall and provides measurement of intima-media thickness (IMT) and presence of plaques [10, 11]. The IMT is significantly higher in diabetic patients than that in nondiabetic patients [12]. And the increased IMT can predict future events of silent brain infarction and coronary heart disease in the patients of type 2 diabetes mellitus [13, 14]. Carotid artery plaque is another marker of systemic subclinical atherosclerosis. But the previous reports showed the inconsistent associations among IMT, plaque, risk factors, and clinical disease [15–18]. And which one is a more powerful predictor of vascular outcomes, IMT or plaque, is still in controversy [18, 19]. In addition, the reported results of the relationship between microalbuminuria and carotid IMT is also different [20, 21]. Therefore, in this study we sought to clarify the relationship between microalbuminuria and markers of carotid atherosclerosis including IMT and plaque in type 2 diabetic patients.

## 2. Materials and Methods

### 2.1. Participants

 The study included 250 patients of type 2 diabetes mellitus at the Department of Endocrinology of the second Affiliated Hospital of Dalian Medical University. Type 2 diabetes mellitus was diagnosed according to the 1999 World Health Organization criteria. The Ethics Committee of the second Affiliated Hospital of Dalian Medical University approved the study. All patients gave their informed consent to participate in the study. According to the level of urinary albumin excretion rate (UAER), 250 patients of type 2 diabetes mellitus were divided into two groups: normoalbuminuria group (UAER < 30 mg/24 h; 130 cases, 66 males and 64 females; mean age, 56.45 ± 9.35 years; age range, 29–76 years; diabetes duration, 7.57 ± 5.53 years; treatment with diet or oral drugs) and microalbuminuria group (30 mg/24 h < UAER < 300 mg/24 h; 120 cases, 62 males and 58 females; mean age, 57.67 ± 11.12 years; age range, 41–80 years; diabetes duration, 8.00 ± 5.12 years; treatment with diet or oral drugs). Medical history was obtained and physical examination was performed in all patients. Blood was withdrawn from all subjects following 12 h of fasting. Type 1 diabetes mellitus, hypertension, history of ischemic heart disease, renal impairment (serum creatinine   > 150 umol/L), and valvular heart diseases were excluded. The clinical conditions that could cause transient elevations in urinary albumin excretion, such as exercise, urinary tract infection, febrile illness, were also excluded.

### 2.2. Carotid Artery Ultrasonography

Carotid artery ultrasonography was performed by an experienced specialist physician who was specifically trained for the vascular ultrasonography. A real-time ultrasound scanner was used: Hitachi EUB 7500 with a linear 3–15 MHz probe (Hitachi Medical Systems, Tokyo, Japan). The patients were examined in the supine position with the head turned 45° contralateral to the side of scanning. B-mode images were obtained in longitudinal section. IMT was defined as the distance between the lumen-intima and the media-adventitia ultrasound interfaces. The IMT on the far wall of the bilateral common carotid artery about 10 mm proximal to the bifurcation of the carotid artery was measured manually as previously described [22, 23]. Three measurements on both sides were performed for each patient and the mean value was obtained for analysis. A high degree of reproducibility (a mean difference in CIMT: 0.020 mm) was shown in paired CIMT measurements in the same arteries. And an intraclass correlation coefficient was 0.93. The presence of plaque was defined as an area of focal wall thickening >50% greater than surrounding wall thickness confirmed by marking and comparing plaque thickness with the thickness of the surrounding wall during scanning by electronic calipers. Furthermore, the plaques were classified into three types: calcified plaques (hyperechogenic), dense plaques (less hyperechogenic than calcified lesions), and soft plaques (isoechogenic in comparison with blood), based on their echogenic properties according to the criteria established by Johnson et al. [24].

### 2.3. Laboratory Assays

The following laboratory parameters were obtained: total cholesterol (TC), triglyceride (TG), low density lipoprotein (LDL), high density lipoprotein (HDL), hemoglobin A1c (HbA1c), fasting plasma glucose (FBG), and urinary albumin excretion rate (UAER). Serum concentrations of TC, TG, LDL, HDL, and FBG were measured by enzymatic method. HbA1C was measured by high performance liquid chromatography (BRO-RAD Company, USA). UAER was obtained by a 24-hour urine collection. Body mass index (BMI) was calculated as weight in kilograms divided by height in meter squared. All the measurements were performed 3 times.

### 2.4. Statistical Analysis

The software of SPSS version 13.0 for Windows (SPSS Inc., IL, USA) was used for statistical analysis. Statistical significance between two groups was determined by the Wilcoxon rank-sum test. Continuous variables were expressed as median and range. Pearson's chi-square (*χ* ^2^) test was used to compare groups regarding categorical variables. Correlation analysis including Pearson's for continuous and Spearman's for discrete variables and multiple linear stepwise regression analysis was used to show the influences of variables on IMT. All tests were performed with *P* < 0.05 considered statistically significant.

## 3. Results

250 type 2 diabetic patients with or without microalbuminuria were screened. The characteristics of the 250 enrolled patients are presented in Table 1. 

**Figure 1:**
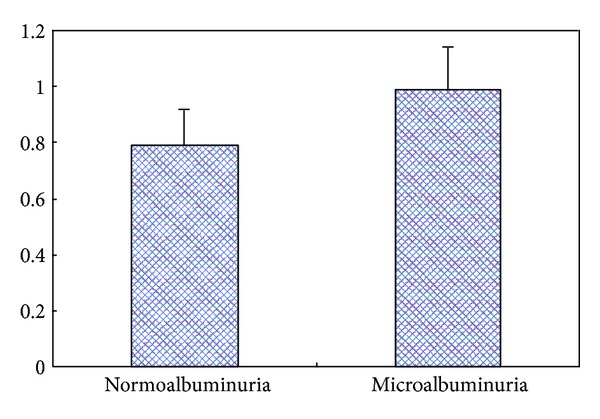
Comparison of IMT between the two study groups. Continuous variables were expressed as median and range. *P* < 0.05 from the Wilcoxon rank-sum test for the difference between normoalbuminuria group and microalbuminuria group. IMT: intima-media thickness.

 Patients of the microalbuminuria group with elevated UAER had higher FBG, BMI, waist and hip circumference, triglycerides, HbA1c, and IMT than those of the normoalbuminuria group with normal UAER (*P* < 0.05 for all; Table 1, Figure 1).

The plaque incidence rate and plaque type rate (soft, dense, and calcified plaques) between two groups were shown in Table 2. The plaque type rate was also compared within the group presented in Table 2. There were no significant difference in plaque incidence rate and plaque type rate between normoalbuminuria and microalbuminuria groups (*P* < 0.05 for all). In both normoalbuminuria and microalbuminuria groups, the rate of soft plaques was the highest. And the rate of soft plaque was higher than that of dense and calcified plaques. The rate of dense plaques was higher than that of calcified plaques. In detail, in normoalbuminuria groups, soft plaques rate (66.67) > dense plaques rate (25) > calcified plaques rate (8.33), and in microalbuminuria group, soft plaques rate (68.42) > dense plaques rate (24.21) > calcified plaques rate (7.37) (Table 2).

In univariate analysis, IMT was positively and significantly associated with age (*r* = 0.265, *P* < 0.05), waist circumference (*r* = 0.263, *P* < 0.05), body mass index (*r* = 0.285, *P* < 0.05), systolic blood pressure (*r* = 0.276, *P* < 0.05), UAER (*r* = 0.359, *P* < 0.05), HbA1c (*r* = 0.462, *P* < 0.05), and duration of diabetes (*r* = 0.370, *P* < 0.05). In multiple stepwise regression analyses, age, body mass index, systolic and diastolic blood pressure, waist and hip circumference, UAER, FBG, plasma HbA1C concentration, serum concentrations of triglycerides and total, HDL and LDL cholesterol, current smoking, and duration of diabetes mellitus were included in the model as independent variables. UAER and HbA1c were appeared to be significantly associated with IMT (*P* < 0.05 for all) (Table 3).

## 4. Discussion

In the present study, we found that the values of IMT of type 2 diabetic patients with microalbuminuria was significantly higher than those without microalbuminuria. And UAER was an independent predictor of IMT. The results indicated that microalbuminuria was related to atherosclerosis in the early stage of diabetic nephropathy. Maybe there is a close relationship between atherosclerosis and diabetic nephropathy. But the mechanism underlying the relationship between microalbuminuria and atherosclerosis in type 2 diabetic patients is still unknown. Nand et al. [25] results showed that microalbuminuria was found to be associated with carotid atherosclerosis in middle aged individuals. But the subjects enrolled in this study were not only limited to the diabetic patients. There was a hypothesis that increased UAER could reflect a generalized vascular dysfunction which was caused by structural alterations, such as a reduction in the density of heparan sulfate-proteoglycan (HS-PG) and/or the sulphation of HS within the extracellular matrix of the glomerular basement membrane and vascular wall [8, 9]. HS-PG is synthesized in endothelial and myomedial cells. It is a normal component of glomerular basement membrane, endothelial vascular surface, and basement membrane of vascular smooth muscle cells. Furthermore, many proteins, such as lipoprotein lipase, tissue factor pathway inhibitor, platelet factor 4, and antithrombin III, are anchored to the vascular wall through interaction with the chains of HS-PG, which may enhance albuminuria and processes involved in atherogenesis [9, 26–28].

Stehouwer et al. [29] found that microalbuminuria was linearly associated with impaired endothelium-dependent, flow-mediated vasodilation in elderly individuals without and with diabetes. It is possible that endothelial leakiness, as reflected by UAE, is in part a primary and possibly genetically determined vascular risk factor, or that it mirrors the endothelial dysfunction featuring the atherosclerotic process or arises from the action of yet unknown risk factors [30]. Also, the previous study results showed that endothelial dysfunction assessed by brachial artery flow-mediated dilation (FMD) was associated with urinary albumin excretion (UAE) and was interrelated with carotid IMT in type 2 diabetic patients with microalbuminuria [31]. 

In the same time, our study showed that the value of HbA1c in type 2 diabetic patients with microalbuminuria was significantly higher than that in patients with normoalbuminuria. And IMT was positively and significantly associated with HbA1c. The result indicated that HbA1c maybe played an important role in the relationship between carotid atherosclerosis and microalbuminuria. HbA1c can accurately reflect longer-term glycemia. Clinically, HbA1c is now used to assess glycemic control in patients of diabetes mellitus, and it is regarded as a useful method of screening and diagnosing diabetes. And HbA1c has been accepted as the best marker for diabetic microvascular complications [30]. Moreover, HbA1c is associated closely with advanced glycation end products (AGEs) [32]. The previous study showed that AGEs are widespread in the diabetic vascular system and contribute to the development of atherosclerosis [33]. AGEs contribute to many microvascular and macrovascular complications through the formation of bridging between molecules in the basement membrane of the extracellular matrix by joining the receptor for advanced glycation end products (RAGE). Concerning microalbuminuria, it was reported that the accumulation of AGEs in the glomerular and tubulointerstitial spaces correlates with the severity of diabetic nephropathy [34]. 

In addition, the results showed that there were no significant difference in plaque incidence rate between normoalbuminuria and microalbuminuria groups. This result was in line with the previous studies [21]. But there was no studies which concerned the plaque types' differences. Our results showed that there were significant differences of plaque types in both normoalbuminuria and microalbuminuria groups. And the incidence rate of soft plaques was the most compared with dense plaques and calcified plaques. It is known that the presence of carotid plaques correlates with an increase in the risk of stroke and cerebral infarction, and softer plaques are more likely to be unstable or vulnerable plaques when compared to calcified plaques [35–38]. 

Our study has its limitations. First, the study population is small. Second, some type 2 diabetic patients have already been treated for diabetes and hyperlipidemia which may lead to inaccuracy of the results.

## 5. Conclusion

Our data show that there is a significant association between microalbuminuria and IMT which is regarded as the early sign of carotid atherosclerosis in type 2 diabetic patients. Routine screening of carotid artery IMT and plaque presence in type 2 diabetic patients with microalbuminuria is necessary. It helps us not only to detect early atherosclerosis but to prevent further development of diabetic nephropathy and cardiovascular events by applying more intensive therapy. However, larger and further studies are needed to confirm our results.

## Figures and Tables

**Table 1:** Characteristics of patients of the study groups (M P_25_−P_75_).

Characteristic	Normoalbuminuria group		Microalbuminuria group		*P* value
	*N* = 130		*N* = 120		
Sex (F/M)	66/64		62/58		NS
Smoker (%)	21.5		22.5		NS
Age (yrs)	50.70	48.82–52.60	51.94	49.71–54.25	NS
Duration of diabetes (yrs)	6.79	5.69–8.03	7.46	6.32–8.38	NS
Waist circumference (cm)	79.88	78.25–81.49	83.25	81.55–84.88	<0.05
Hip circumference (cm)	85.16	83.84–86.23	89.6	88.2–102.5	<0.05
BMI (kg/m^2^)	23.34	22.65–24.25	25.55	25.02–26.37	<0.05
Systolic BP (mmHg)	116.48	13.38–119.77	117.14	113.65–120.34	NS
Diastolic BP (mmHg)	74.92	73.5–76.7	73.65	71.76–75.45	NS
FBG (mmoL)	8.38	7.55–9.25	11.65	10.62–12.64	<0.05
HbA1c (%)	6.54	6.31–6.82	7.92	7.72–8.15	<0.05
Total cholesterol (mmol/L)	4.46	4.22–4.70	4.66	4.52–4.88	NS
Triglycerides (mmol/L)	1.52	1.26–1.78	2.12	1.85–2.39	<0.05
LDL cholesterol (mmol/L)	3.05	2.79–3.29	3.15	2.97–3.30	NS
HDL cholesterol (mmol/L)	0.92	0.84–0.97	0.92	0.86–0.96	NS
UAER (mg/24 hr)	11.25	9.49–12.8	87.55	74.56–100.58	<0.05
IMT (mm)	0.75	0.72–0.77	0.92	0.87–0.95	<0.05

NS: not significant; F/M: female/male; BP: blood pressure; BMI: body mass index; FBG: fasting blood glucose; LDL: low density lipoprotein; HDL: high density lipoprotein; UAGR: urinary albumin excretion rate.

**Table 2:** Comparisons of plaques' types and rates of two groups.

Groups	Presence of plaques (cases)			Plaques' types (number and rate)		
	Cases	+	−	Soft plaques	Calcified plaques	Dense plaques
Normoalbuminuria group	130	69	61	72 (66.67)	9 (8.33)	27 (25)
Microalbuminuria group	120	62	58	65 (68.42)	7 (7.37)	23 (24.21)

Comparison of plaques' rates between two groups: *χ* ^2^ = 0.209, *P* > 0.05; comparison of plaques' types and numbers between two groups: *χ* ^2^ = 0.210, *P* > 0.05.

**Table 3:** Multiple stepwise regression analyses.

Characteristics	IMT	
	Standardized coefficient beta	*P* value
UAER (mg/24 hr)	0.269	<0.05
HbA1c (%)	0.458	<0.05

The model including age, body mass index, systolic and diastolic blood pressure, waist and hip circumference, UAER, FBG, plasma HbA1C concentration, serum concentrations of triglycerides and total, HDL and LDL cholesterol, current smoking, and the duration of diabetes mellitus.
